# Left Ventricular Hypertrophy in Diabetic Cardiomyopathy: A Target for Intervention

**DOI:** 10.3389/fcvm.2021.746382

**Published:** 2021-09-29

**Authors:** Mohapradeep Mohan, Adel Dihoum, Ify R. Mordi, Anna-Maria Choy, Graham Rena, Chim C. Lang

**Affiliations:** ^1^Division of Mental Health and Wellbeing, Warwick Medical School, University of Warwick, Coventry, United Kingdom; ^2^Division of Molecular and Clinical Medicine, School of Medicine, Ninewells Hospital and Medical School, University of Dundee, Dundee, United Kingdom; ^3^UKM Medical Molecular Biology Institute (UMBI), Universiti Kebangsaan Malaysia, Kuala Lumpur, Malaysia

**Keywords:** diabetic cardiomyopathy (DCM), heart failure, type 2 diabetes mellitus, metformin, allopurinol, SGLT2 inhibitors, left ventricular hypertrophy (LVH)

## Abstract

Heart failure is an important manifestation of diabetic heart disease. Before the development of symptomatic heart failure, as much as 50% of patients with type 2 diabetes mellitus (T2DM) develop asymptomatic left ventricular dysfunction including left ventricular hypertrophy (LVH). Left ventricular hypertrophy (LVH) is highly prevalent in patients with T2DM and is a strong predictor of adverse cardiovascular outcomes including heart failure. Importantly regression of LVH with antihypertensive treatment especially renin angiotensin system blockers reduces cardiovascular morbidity and mortality. However, this approach is only partially effective since LVH persists in 20% of patients with hypertension who attain target blood pressure, implicating the role of other potential mechanisms in the development of LVH. Moreover, the pathophysiology of LVH in T2DM remains unclear and is not fully explained by the hyperglycemia-associated cellular alterations. There is a growing body of evidence that supports the role of inflammation, oxidative stress, AMP-activated kinase (AMPK) and insulin resistance in mediating the development of LVH. The recognition of asymptomatic LVH may offer an opportune target for intervention with cardio-protective therapy in these at-risk patients. In this article, we provide a review of some of the key clinical studies that evaluated the effects of allopurinol, SGLT2 inhibitor and metformin in regressing LVH in patients with and without T2DM.

## Introduction

**Figure 1:**
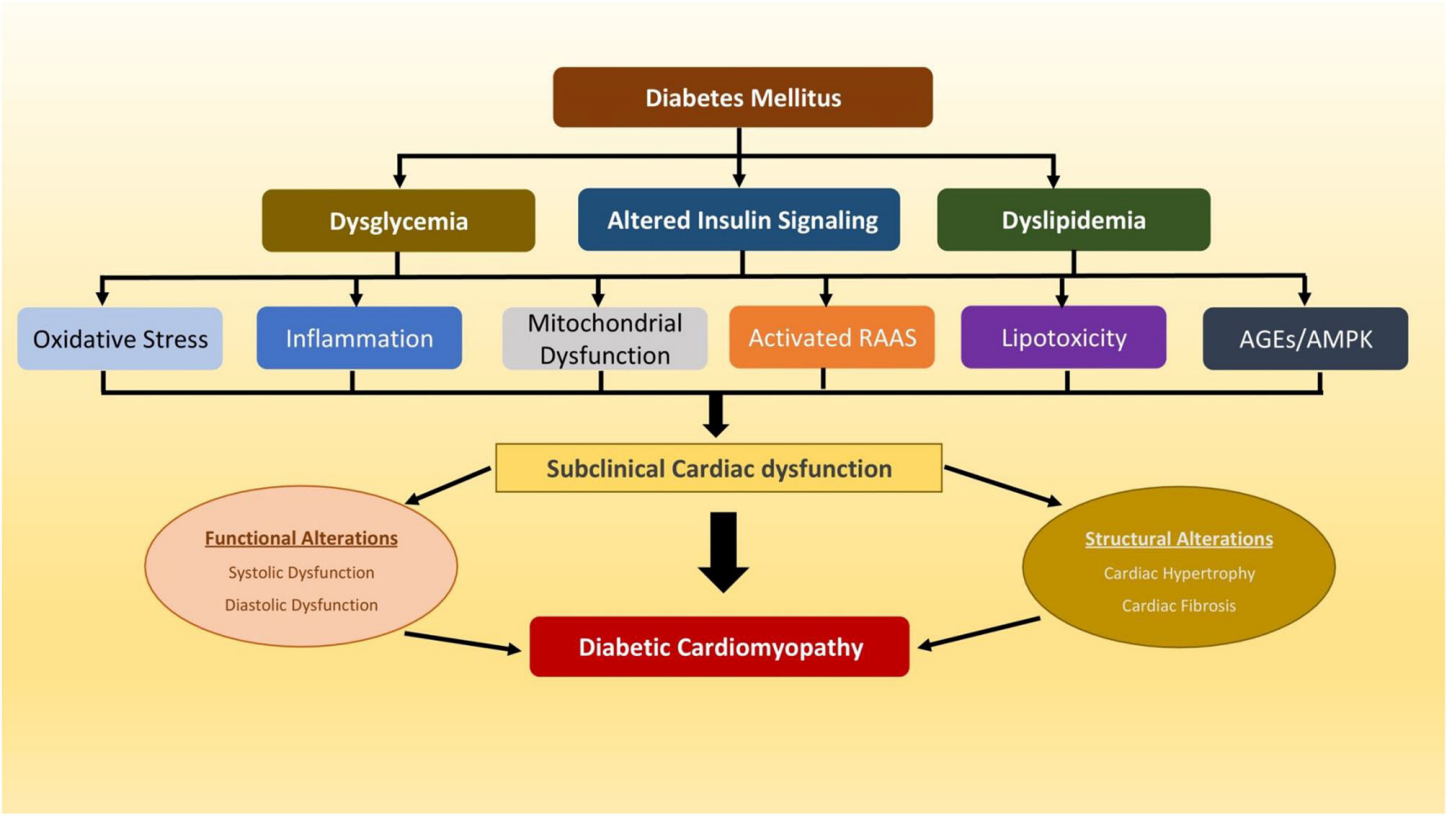
A schematic diagram depicting multiple potential mechanisms that have been implicated in pathophysiology of diabetic cardiomyopathy. AGEs, advanced glycation end products; AMPK, AMP-activated protein kinase; RAAS, renin-angiotensin-aldosterone system.

Diabetic cardiomyopathy (DCM) is defined as cardiac dysfunction, characterised by abnormal structural, functional and metabolic changes in the myocardium, that occurs in the absence of significant coronary, valvular or hypertensive diseases in individuals with diabetes (1). Diabetic cardiomyopathy was first described five decades ago (2), and the higher incidence of heart failure (HF) in patients with type 2 diabetes mellitus (T2DM) was further confirmed in several epidemiological studies, including the Framingham Heart Study (3–5). The aetiology of DCM in the diabetic heart is complex and is likely to multifactorial (Figure 1). In the early stages of its progression, DCM is usually asymptomatic and is characterised by subclinical structural and functional abnormalities including left ventricular hypertrophy (LVH), reduced LV compliance, myocardial fibrosis and stiffness (6). These pathophysiological changes associated with subclinical cardiac dysfunction can be progressive and lead to HF symptoms and to the clinical syndrome of HF (7). Despite the success of various antihyperglycemic agents in the intensive management of hyperglycemia in people with T2DM in reducing the risk of cardiovascular (CV) complications, the high prevalence of HF persists (8–10), that might suggest the role of other non-hyperglycemia associated pathophysiological mechanisms that might contribute to the development of DCM and consequent higher risk of HF in T2DM.

LVH is defined by an elevated left ventricular mass (LVM), either due to an increase in wall thickness or due to left ventricular cavity enlargement, or both. Typically, the LV wall thickening manifests in response to hemodynamic pressure overload, and chamber dilatation occurs in response to volumetric burden, with the caveat that other molecular and metabolic mechanisms may play a synergistic and potentially independent role. Importantly, LVH is strong predictor of CV events (11, 12), and is a common feature in the diabetic myocardium (7, 13). The reason why LVH is predictive of CV events is because it precedes many potentially fatal CV sequelae of events. For instance, the presence of LVH can lead to adverse cardiovascular (CV) outcome—LVH impedes left ventricular (LV) filling and can lead to diastolic heart failure; it reduces coronary perfusion reserve and can induce ischemia; it can lead to left atrial enlargement and subsequent atrial fibrillation and it is intrinsically arrhythmogenic and can cause sudden cardiac death (14). The development of LVH in T2DM is not fully explained by the hyperglycemia-associated cellular alterations alone (15). Specifically, there is extensive body of clinical and experimental underpinnings that supports the role of inflammation, oxidative stress, insulin resistance and AMP-activated kinase (AMPK) in mediating the development of LVH in T2DM (15–18). While the pathophysiology of LVH in diabetes is not yet fully elucidated, the increasing recognition that LVH is an exquisite orchestration of a wide array of pathophysiological process, that are not limited to hyperglycemia, hypertension and valvular disease, provided new opportunities to examine new pharmacological therapies in LVH regression.

As defined by the American College of Cardiology/American Heart Association (ACC/AHA) guidelines, HF progresses as a clinical continuum of four stages (19). Stage B HF represents patients with structural heart disease including LVH, but with no current or prior symptoms of HF (19). To prevent progression to symptomatic HF (Stage C HF), it is important to identify the presence of DCM at the early stages of its development. While the electrocardiogram (ECG) is a widely used method to diagnose LVH, its diagnostic accuracy is limited due to its poor sensitivity in detecting LVH (20, 21). Echocardiography has been conventionally considered a test of choice to evaluate the presence of cardiac dysfunction and early structural changes of the heart including LVH (22). Its sensitivity is significantly higher than ECG in detecting LVH (23), and can also diagnosis other subclinical cardiac abnormalities and valvular heart disease. However, cardiac magnetic resonance imaging is considered the gold standard due to high precision and reproducibility in estimating LV mass (24) and other structural cardiac abnormalities, albeit its use is limited due to its high costs and limited availability.

In addition to multiple comorbidities and several molecular and metabolic mechanisms, several studies have reported that LVH is also influenced by various other factors such as age, gender, ethnicity and genetic factors. For instance, LVH is more prevalent in blacks compared with other race/ethnic groups (25, 26). The prevalence of LVH is not reported to be dissimilar between men and women, irrespective of diagnostic criteria applied (27). The prevalence of LVH also increased significantly with age, occurring in 33% of men and 49% of women over 70 (28). Furthermore, genome wide association studies (GWAS) have also reported substantial heritability for LVH in diverse populations including diabetic patients (29, 30).

In this mini-review, we summarise the epidemiology of LVH in T2DM and the key pathophysiological mechanisms and provide a review of some of the key studies that evaluated the effects of different classes drugs on LVH regression.

## Left Ventricular Hypertrophy in Diabetic Cardiomyopathy

### Prevalence and Economic Impact

It is becoming increasingly recognised that T2DM is an independent risk factor for LVH, even in the absence of overt cardiovascular diseases (CVD) (31, 32). T2DM is often associated with cardiomyopathy, manifested by LVH, and the reported prevalence of LVH in patients with T2DM ranges from 32 to 71% in different studies, depending on the criteria used for defining LVH (12, 33–36). Importantly, previous research has demonstrated that LVH regression *per se* independently reduces CV mortality and events (37, 38). Thus, routine screening of LVH, followed by targeted intervention could be a promising way of reducing CV events and mortality in people with T2DM. Furthermore, disability caused by CVD carries major health economic consequences at both individual levels, and the society as a whole, and this burden is expected to increase in the future. Therefore, routine screening of LVH and targeted treatment may have the potential to reduce the economic burden of CVD. While it is difficult to evaluate the economic value of saving life, the costs of screening for and treating LVH is anticipated to be low, primarily due to the high prevalence of the condition and the low unit cost of echocardiography (39).

### Efficacy and Cost-Effectiveness of Screening

The efficacy and cost effectiveness of early screening for traditional CV risk factors has already been proven in the general population (40–42). However, an ongoing challenge is to develop adequate screening strategies that are cost-effective that can identify individuals at risk for future CV events, prior to developing overt CVD. Since people with T2DM are at a higher risk for future CV events than non-diabetic people, it may be more cost-effective to target subclinical cardiac abnormalities including LVH in diabetic population. Whilst this approach may initially increase healthcare costs, especially with the costs associated with CV imaging and treatment, such an approach may in the longer term reduce CV related mortality and morbidity in patients with T2DM. Witham et al. in a hypothetical analysis of the cost-effectiveness of screening LVH, suggested that this may indeed be a very cost-effective strategy to reduce CV events in high-risk people including those with T2DM (39). Biomarkers may prove to be an alternate tool that is even more cost-effective, when used in CVD risk prognostication (43). In this context, previous studies have utilised a biomarker-based approach for the identification of subclinical LV abnormalities in patients without CVD. For instance, in patients with T2DM and without HF, higher levels of B-type natriuretic peptide (BNP) associated with LVH and lower left ventricular ejection fraction (44). Another study of non-diabetic individuals without overt CVD, found that high sensitivity cardiac troponin T (hs-cTnT) and BNP may be suitable for (the c-statistic for screening with BNP ± hs-cTnT was 0·81) identifying asymptomatic cardiac diseases including LVH (45). If the results of these studies can be confirmed in future large studies, pre-screening of people with T2DM using the biomarker approach may offer a cost-effective modality in screening LVH. Further systematic health economics studies are warranted to assess the cost-effectiveness of screening T2DM patients for LVH, and then pre-emptive treatment.

### Pathophysiology of LVH and Potential Targets

#### Inflammation

Inflammation is a prominent hallmark of LVH. Pro-inflammatory cytokines are found to modulate left ventricle LV structure and play a critical role in LV remodelling (46). Inflammatory mediators can result in changes in cardiac size, shapes, and composition, including myocyte hypertrophy, alterations in expression of foetal gene, and progressive myocyte loss through apoptosis (46). Pro-inflammatory cytokines such as IL-1, IL-6 and tumour necrosis factor-alpha (TNF-α) are normally not expressed in the heart, but they are activated and up regulated as response to myocardial injury or mechanical stress, contributing to cardiac remodelling (46). Soon after ischemic myocardial injury, TNF-α and IL-6 are elaborated and trigger additional cellular inflammatory responses (47). Chronically, to repair and remodelling, these cytokines activate matrix metalloproteinases and collagen formation, and regulate angiogenesis and progenitor cell mobilisation, resulting in development of myocardial hypertrophy, collagen deposition, and fibrosis (47). In macrophages, lipopolysaccharide (LPS), tumour necrosis factor-α, and interferon-γ, has been shown to be implicated in cardiac hypertrophy via their effects on the expression of microRNA-155 (miR-155) expression, a powerful mediator of cardiac hypertrophy (48). Notably, variable myocyte hypertrophy and inflammatory cell infiltration with activation of nuclear factor kappa B (NF-κB) have been identified in endomyocardial tissue of patients with Hypertrophic cardiomyopathy (HCM) while the levels of in interleukins (IL-1β, IL-1RA, IL-6, IL-10) and high-sensitivity C-reactive protein (hsCRP) have been found to be significantly higher in patients with HCM than in control subjects (49). These suggest that a low-grade inflammatory response may play an important role in the development of cardiac hypertrophy in patients with HCM and support the causative significance of inflammatory signalling in hypertrophic heart disease, demonstrating the feasibility of therapeutic targeting of inflammation in heart failure.

#### Oxidative Stress

Growing evidence points to the potential involvement of oxidative stress in the pathophysiology of cardiac hypertrophy *via* different possible mechanisms. Dysregulated expression of nitrogen oxides (NOX) proteins, which are predominant isoforms expressed in cardiac tissue, has been proposed to contribute to the development of cardiac hypertrophy and is believed to driven by eroxisome proliferator-activated receptor α (PPARα) downregulation (50). Upregulation of NOX4 by angiotensin II, which is primary in mitochondria of cardiomyocytes, can also result in inducing nuclear export of histone deacetylase 4 (HDAC4), a crucial precursor of cardiac hypertrophy (51). NOX2 and NOX4 have also been found to be associated with cardiac hypertrophy and fibrosis in diabetic rats, and the elevation of NOX2 has been shown to be associated with an increase in cardiomyocytes size in mice subjected to high fat diet (50). Furthermore, In an experimental guinea pig model of pressure-overload induced cardiac hypertrophy, increased oxidative stress can result in an increase in cardiac ROS production, which induces cardiac hypertrophy through activation of redox-sensitive protein kinases such as mitogen activated protein kinase (MAPK) (52). In neonatal cardiomyocytes, however, ROS has been found to active a wide variety of hypertrophic signalling kinases and transcription factors, including the tyrosine kinase Src, GTP-binding protein Ras, protein kinase C, mitogen-activated protein kinases (extracellular response kinase and extracellular signal–regulated kinase), and Jun-nuclear kinase, NK-KB and Phosphoinositol 3-kinase, which is required for H2O2-induced hypertrophy (53). Finally, in diabetic patients, it is hypothesised that myocardial kinases β isoform of protein kinase C (PKCβ), which is preferentially overexpressed in diabetic myocardium accompanied with increased upregulation of pro-oxidant enzyme NAPH oxidase, that is a major upstream moderator of oxidative stress and that inhibition of PKCβ can attenuate myocardial hypertrophy (54). All these observations support the role of oxidative stress in the pathophysiology of cardiac hypertrophy.

## Role of Insulin Signalling, mTOR, and AMPK in Cardiac Hypertrophy

Insulin resistance has long been recognised as a common factor in and contributing towards the intersection between diagnosis of heart disease and diabetes in the same patient (55). In brief, development of insulin resistance over time, initially compensated by hyperinsulinemia, but ultimately resulting in beta cell failure, likely results in successive periods where there is first overstimulation and then under stimulation of anabolic insulin-sensitive signalling pathways in cardiomyocytes and other tissues, any of which, as well as the metabolic flexibility inherent in insulin resistance, may contribute to pathology. Many of the proteins mediating insulin signalling are encoded by more than one gene, which has complicated mechanistic genetic analysis. Consistent with the likely complex role of insulin signalling in heart disease, deletion of IRS1 and IRS2 docking proteins in liver results in heart failure in mice, whereas deletion of the same genes in cardiac tissue resulted in smaller ventricular mass (56). These findings also illustrate that insulin signalling in cardiac tissue and in non-cardiac tissue, are both likely to have an important impact on progression of heart disease. More work is required to determine the critical metabolic regulatory pathways underlying these effects.

One of the metabolic signalling pathways most studied in cardiac dysfunction is mammalian target of rapamycin (mTOR) signalling. The mTOR signalling pathways are at the crossroads of anabolic and catabolic cellular process (57). mammalian target of rapamycin complex 1 (mTORC1) promotes anabolic metabolism in response to growth factor signalling, nutrients (particularly amino acids), and increased energy supply. mTORC1 also suppresses autophagy (58). mTORC1 phosphorylates Unc-51 like autophagy activating kinase (ULK1), a kinase that forms a complex with ATG13, FIP2000, and ATG101 and drives autophagosome formation, disrupting the interaction between Ulk1 and AMPK and prevent Ulk1 activation by AMPK (59). At transcriptional level, mTORC1 can also regulate autophagy. It prevents nuclear transport of the transcription factor EB (TFEB), which drives genes expression for lysosomes biogenesis and the autophagy machinery (60). In these ways mTORC1 is understood largely to determine the extent of cellular anabolic and catabolic processes, whilst in contrast mTORC2 seems to function mainly as an effector of insulin/IGF-1 signalling ref 8. mTORC2 activates and phosphorylates protein kinase B (PKB), a key effector of insulin/PI3K signalling. Phosphorylation of PKB results in cell promotion and proliferation via activation and suppression of many key substrates including the transcription factors of FoxO1/3a, the metabolic regulator (GSK3β), and the mTORC1 inhibitor (TSC2) (58). mTORC2 is also regulated by mTORC1 through a negative feedback loop between mTORC1 and insulin PI3K signalling. mTORC1 activates (Grb10), a negative regulator of insulin/IGF-1 receptor signalling upstream of Akt and mTORC2 (58).

In the cardiovascular system, mTOR pathways are understood to be important regulators of the hypertrophic response (61). Loss of function genetic studies indicate that mTORC1 activation is indispensable for development of cardiac hypertrophy in response to pressure overload. An animal study showed that the deletion of Rheb 1, which mediates mTORC1 activation, in cardiomyocytes confers cardioprotection against pathological remodelling in pressure overload (62). The mTORC1 regulates cardiac function and myocyte survival through 4E-BP1 inhibition in mice. A study showed a significant improvement in apoptosis and heart function when Mtor and the gene encoding 4E-BP1, an mTOR-containing multiprotein complex-1 (mTORC1) substrate that inhibits translation initiation, was deleted together (63).

Furthermore, in mice, ablation of cardiac raptor, a gene required for normal cardiac physiological function and for heart adaptation to increased workload, is shown to impair adaptive hypertrophy, change metabolic gene expression, and causing heart failure (64). In raptor-cKO mice, pressure overload causes a significant cardiac dilatation and immediate reduction in ejection fraction (64).

Consistent with this, pharmacological inhibition of mTORC1 also suppresses hypertrophy. Rapamycin, a specific inhibitor of mTOR, inhibits established cardiac hypertrophy via a complete suppression the phosphorylation of S6K1 and S6 phosphorylation in response to pressure overload (65). This can explain the attenuation of the increase in myocyte cell size induced by aortic constriction after administering Rapamycin to mice (66). Rapamycin is also shown to extend lifespan of mice (67). However, although it is shown that rapamycin can reverse age-dependent defects in cardiac function (68) and can protect against the changes of atherosclerosis (69), it is still unclear if an increase in lifespan owes to effects on cardiovascular system (67).

AMPK is another protein kinase that regulates many aspects of cellular energy balance and is seen as a promising target for anti-hypertrophic drugs. AMPK activation reverses increased protein O-GlcNAcylation, which is associated with cardiac hypertrophy, mainly through controlling the glutamine: fructose-6-phosphate aminotransferase (GFAT) phosphorylation, resulting in a decrease in O-GlcNAcylation of proteins such as troponin T (70). In line with this, an increase in cellular O-GlcNAc levels in response to O-linked N-acetylglucosamine (O-GlcNAc) inducers completely supresses the anti-hypertrophic effect of AMPK (70).

Metformin and AICAR have each been shown in preclinical studies to inhibit cardiac hypertrophy. Long-term metformin treatment significantly increases AMPK phosphorylation and attenuates cardiac hypertrophy induced by Transverse aortic constriction (TAC) (71). Interestingly, the antihypertrophic effects of metformin were not observed in AMPKα2–/– mice (71), suggesting that the chronic activation of AMPK during the development of cardiac hypertrophy is an important mechanism that mediates the beneficial effect of metformin. Moreover, long-term activation of AMPK by AICAR has been shown to block load-induced calcineurin–NFAT pathway as calcineurin is also regulated by MAPK pathway. Thus, the activation of AMPK may counteract MAPK pathway though blocking calcineurin–NFAT pathway (72).

However, counterbalancing these promising findings is recent evidence that a pan-AMPK activator improves glucose homeostasis, but at the cost of increased hypertrophy. Chronic activation of MK-8722, pan-AMPK activator, increases glucose uptake into skeletal muscle, but it results in cardiac hypertrophy associated with increased cardiac glycogen contents (73). To determine which of these beneficial and less beneficial aspects of the drugs really do owe to AMPK, further genetic studies are required.

### Role of Drug Repurposing in LVH Regression

Due to the high attrition rates, significant economic burden and lengthy new drug discovery process, repurposing of ‘old’ drugs (also known as drug repositioning, reprofiling or re-tasking) to treat both common and rare diseases is increasingly becoming a promising field in drug discovery. Drug repurposing identifies new therapeutic uses for already approved drugs that are outside the scope of the original use (74). This is an attractive proposition because it involves the use of existing compounds that have undergone rigorous testing with potentially lower overall development costs and shorter development timelines.

Hypertension is the most common causes of LVH and therefore, controlling blood pressure (BP) especially with drugs that blocks the renin-angiotensin system (RAS) is the standard approach to the management of LVH. However, this approach is only partially effective since LVH persists in 20% of hypertensives that attain target BP (75). Thus “normotensive LVH” is very common (12). Indeed, BP only contributes 25% to the variability in LV mass seen in a population (76). Despite a “normal” BP, normotensive LVH is just as risky as is hypertensive LVH (77). Nevertheless, regressing LVH irrespective of BP changes is an effective way to reduce the incidence of all major CV events including specifically sudden deaths, heart failure hospitalisations, new onset AF and strokes (37, 38, 78–83). The LIFE study demonstrates that LVH regression *per se* reduces future cardiovascular events irrespective of BP (84). Since controlling BP and using a RAS blocker is only partially effective at regressing LVH, we now need additional ways of regressing LVH. In addition to BP, as we have discussed above, the pathophysiology of LVH may involve a complex cocktail of various non-hemodynamic disease processes including inflammation, oxidative stress, obesity, and insulin resistance (15, 29, 31, 53, 85–90).

Below, we provide a narrative review of some of the key clinical trials that evaluated the effects of interventions targeting LVH in patients with and without T2DM, in the context of normal BP.

### Targeting LVH With Allopurinol

Allopurinol, a xanthine oxidase (XO) inhibitor, has been the mainstay of treatment for patients with gout associated with hyperuricemia for several decades. In addition, there is mounting evidence to suggest cardioprotective effects of allopurinol (91–93). Allopurinol has been shown to exert its cardioprotective effects through three key mechanisms: (i) reduction of uric acid concentrations which has pro-inflammatory effects; (ii) inhibiting xanthine oxidase mediated production of reactive oxygen species (ROS) which aggravate endothelial dysfunction and atherosclerosis plaque instability; and (iii) increasing local tissue availability of adenosine triphosphate and oxygen by inhibiting purine metabolism (94).

Allopurinol has been shown to regress LVM in different cohorts along the cardiovascular spectrum with significant pre-existing disease, oxidative stress and inflammation. In a RCT of people with T2DM, 9 months treatment of allopurinol treatment significantly reduced absolute left ventricular mass (LVM) (allopurinol −2.65 ± 5.91 g vs. placebo group +1.21 ± 5.10 g; *p* = 0.012) and LVM indexed (LVMI) to body surface area (allopurinol −1.32 ± 2.84 g/m(2) vs. placebo group +0.65 ± 3.07 g/m(2); *p* = 0.017) (18). In another RCT of people with ischemic heart disease, allopurinol treatment significantly reduced LVM (allopurinol −5.2 ± 5.8 g vs. placebo −1.3 ± 4.48 g; *p* = 0.007) and LVMI (allopurinol −2.2 ± 2.78 g/m(2) vs. placebo −0.53 ± 2.5 g/m(2); *p* = 0.023) (17). Furthermore, Kao et al., in another RCT of patients with severe renal disease (CKD 3) reported significant reduction in LVM with 9 months allopurinol treatment (95). In contrast, a recent study by Gingles et al., reported that LVM regression was significantly reduced with allopurinol treatment than placebo, suggestive of a potential adverse effect (96). However, unlike other studies, in this RCT, the study population had well-controlled BP and were normouricemic with low oxidative stress. This may have negated any direct effect of allopurinol, who rely on urate for antioxidant defence, in reducing ROS generation by XO inhibition, and consequent null effect on LVH. Therefore, the regressive effect of allopurinol may not be observed universally in all study populations.

#### Targeting LVH With Metformin

Metformin is an oral antihyperglycemic agent that has been used widely for the treatment of T2DM for over many decades. Beyond its antihyperglycemic effects, there is now accumulating evidence to suggest that metformin is cardioprotective (97). While the exact mechanism of cardioprotective actions of metformin is not fully understood, several ancillary mechanisms have been proposed to explain the metformin induced LVH regression. First, as stated already, insulin resistance, inflammation, oxidative stress, endothelial dysfunction and obesity is understood to contribute to the development of LVH (15, 53, 85–89), and metformin has been shown to reduce insulin resistance (98), inflammation (99), oxidative stress (100–104), central obesity (105) and endothelial dysfunction (106), albeit the latter is not a consistent finding (107). Second, *in vivo* studies have reported activation of AMP-activated protein kinase (AMPK) as one of the putative mechanisms for the anti-hypertrophic effect of metformin (15), leading to the view that AMPK stimulation is a promising new strategy to prevent or reduce LVH (108–110).

For clinical trials, to date, only one study: The MET-REMODEL trial, has explicitly investigated the effect of metformin on LVH in non-diabetic CAD patients identified to have IR and/or diabetes. This study demonstrated that 12 months metformin treatment (2 g/day) significantly reduced LVMI (absolute mean difference −1.37 (95% CI: −2.63 to −0.12, *P* = 0.033) (111). In this study, metformin also significantly reduced LVM, systolic blood pressure, and oxidative stress. In line with these findings, few other clinical studies and a network analysis also reported anti-hypertrophic effects of metformin (112–114).

### Targeting LVH With SGLT2 Inhibitors: Multipronged Approach

The sodium-glucose linked cotransporter type 2 (SGLT2) class of inhibitors was developed as a novel anti-diabetic agent that acts independent of the insulin-incretin pathway to lower blood sugar. Various classes of SGLT2 inhibitors such as empagliflozin, canagliflozin and dapagliflozin have been shown to reduce cardiovascular mortality in patients with diabetes mellitus (115–118), but its cardioprotective mechanism remains elusive. More recent evidence from RCTs, suggest the potential of dapagliflozin in reducing the risk of worsening heart failure or CV mortality, even in non-diabetic population (119, 120). However, it is not clear whether the cardioprotective effects of SGLT2 inhibition in non-diabetic patients is class effect or drug-specific effect (121–124). Unlike other antidiabetic agents that are dependent on pancreatic beta-cell function and insulin sensitivity for the glucose lowering effect, the principal mechanism by which SGLT2 inhibitors lower blood glucose is by excreting excess glucose by enhancing urinary glucose excretion. Part of the off-target effects that have been observed with this class of drugs include weight loss, improved glycemia/lipid profile, arterial stiffness, reduce preload and afterload (blood pressure) and diuresis (125, 126)—all of which are key risk factors implicated in the development of LVH (15).

The DAPA-LVH study was the first placebo controlled RCT to investigate the efficacy of dapagliflozin in regressing LVH in normotensive patients with T2DM, without pre-existing CVD (127). At 12 months, dapagliflozin treatment significantly reduced LVM in people with T2DM, as assessed by cardiac magnetic resonance imaging (128). In this study, dapagliflozin was also shown to significantly reduce systolic BP, body weight, abdominal obesity (both visceral and subcutaneous), insulin resistance, and hsCRP. A similar finding was observed in the EMPA-HEART study that reported anti-hypertrophic effect of empagliflozin (129). In the DAPA-LVH trial, LVH regression was greater in those with higher baseline LVM (128). It is to be noted that a recent subgroup analysis of the EMPA-REG OUTCOME trial reported lower incidence of CVD in patients with LVH compared with those without LVH (130). Furthermore, *post-hoc* exploratory analysis from DAPA-LVH trial suggest dapagliflozin may improve subclinical dysfunction, as evidenced by improved myocardial longitudinal function (131). Taken together, there is compelling evidence to suggest that SGLT2 have the potential to promote reverse LV remodelling in patients with diabetes, which may, at least in part, explain the cardioprotective effects observed in large outcome trials of SGLT2.

## Conclusions

**Figure 2:**
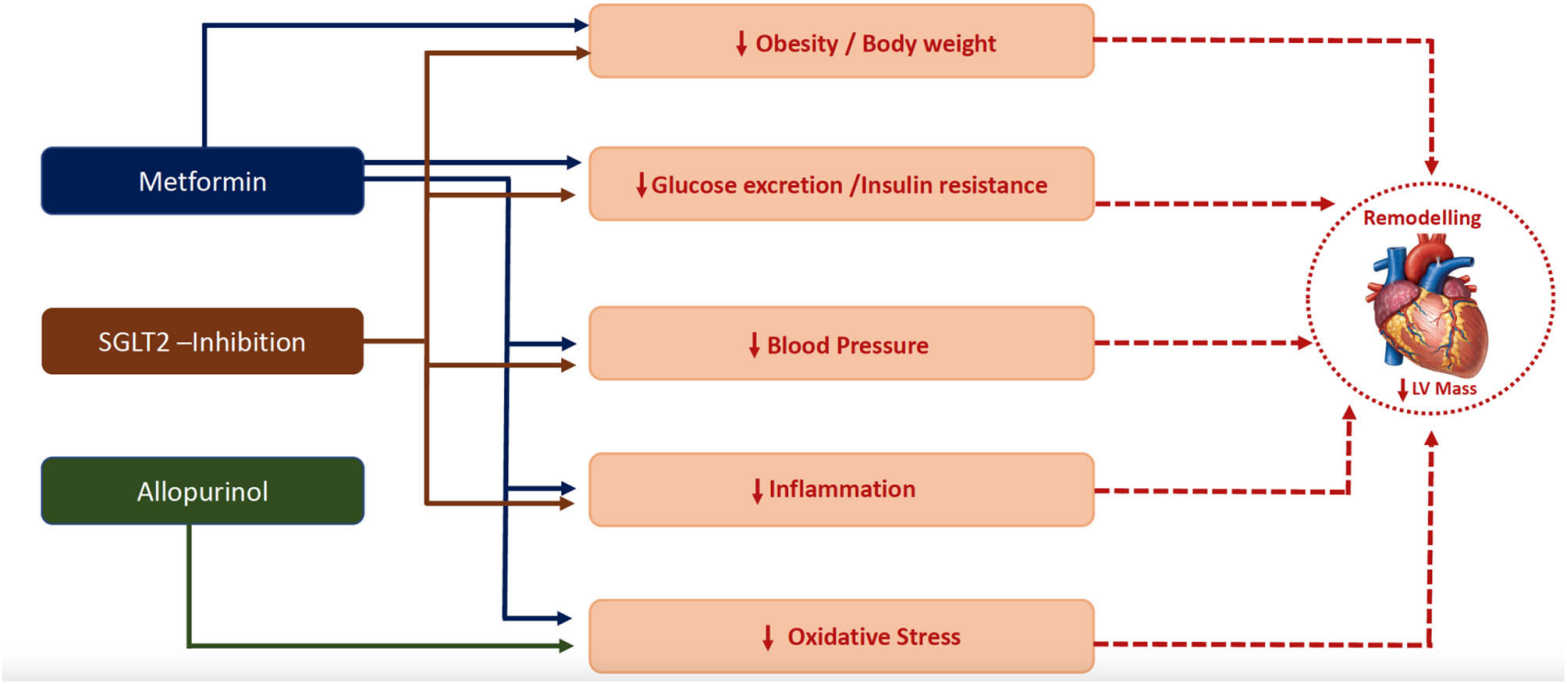
Plausible mechanisms by which metformin (111), SGLT-2 inhibitor (128) and allopurinol (17, 18) regressed left ventricular hypertrophy.

In this mini-review, we have argued that LVH is a good surrogate marker of diabetic cardiomyopathy and discussed the trials targeting LVH as a manifestation of Stage B Cardiomyopathy, and potential mechanisms behind LVH regression (Figure 2) in patients with T2DM or in insulin-resistant individuals. We believe that cardiovascular outcome trials are still needed to provide definitive evidence for the cardio-protective role of the proposed repurposed drugs. With respect to metformin, the results of the on-going outcome trials such as the VA IMPACT trial (VA IMPACTNCT02915198) and Glucose Lowering in Non-diabetic hyperglycaemia Trial (GLINT; ISRCTN34875079), will be informative and might provide the needed evidence for recommending metformin in these at risk patients.

## Future Research

All the clinical studies discussed in this manuscript were “proof of concept” studies, conducted in small sample size, to evaluate the possible mechanisms behind the purported cardio-protective effects of each drugs. Unlike, the LVH regression observed in previous hypertension trials, the magnitude of the LVH regression observed in these trials were small, which may be, at least in part due to the shorter follow-up period and treatment duration. The results of these proof-of-concept trials are encouraging and help underpin.future large cardiovascular outcome trials, with longer follow-up period, incorporating better hard end points. One such trial is the VA-IMPACT trial (VA IMPACT NCT−02915198) of metformin. Such trials will be informative and help provide the medical evidence to support the use these drugs in diabetic cardiomyopathy.

## Author Contributions

CCL and MM conceived the idea. MM designed the figure. MM and AD wrote the first draft of the manuscript and was responsible for synthesizing the evidence, the search strategy, and conducted the literature search. IM, AMC, GR, and CCL critically reviewed and revised the manuscript. All authors read and approved the final manuscript.

## Conflict of Interest

The authors declare that the research was conducted in the absence of any commercial or financial relationships that could be construed as a potential conflict of interest.

## Publisher's Note

All claims expressed in this article are solely those of the authors and do not necessarily represent those of their affiliated organizations, or those of the publisher, the editors and the reviewers. Any product that may be evaluated in this article, or claim that may be made by its manufacturer, is not guaranteed or endorsed by the publisher.
